# Ploidy Level Influences Pollen Tube Growth and Seed Viability in Interploidy Crosses of *Hydrangea macrophylla*

**DOI:** 10.3389/fpls.2020.00100

**Published:** 2020-02-19

**Authors:** Lisa Alexander

**Affiliations:** Floral and Nursery Plants Research Unit, Otis L. Floyd Nursery Research Center, USDA-ARS, United States National Arboretum, McMinnville, TN, United States

**Keywords:** pollen tube, polyploid, mating barrier, triploid, hydrangea, ornamental plant breeding

## Abstract

All *Hydrangea macrophylla* cultivars tested to date are diploid or triploid and triploid *H. macrophylla* have thicker stems, larger flowers, and larger stoma compared to related diploids. It is unknown whether interploidy crosses between diploid and triploid hydrangeas can be used to develop triploid varieties. The objective of this study was to compare pollen tube development, fruit formation, and seed viability among intra- and interploidy pollinations of *H. macrophylla* and evaluate the genome size and pollen viability of resultant progeny. By 24 h post-pollination, pollen tubes had reached the ovaries of diploid flowers in 48.7% of samples while pollen tubes reached the ovaries in only 8.7% of triploid flowers (*χ* ^2^ = 30.6, *p* < 0.001). By 48 h post-pollination pollen tubes reached the ovaries of diploid and triploid flowers in 72.5% and 53.8% of samples, respectively (*χ* ^2^ = 26.5, *p* = 0.001). There was no difference in percentage of flowers with pollen tubes reaching the ovaries in diploid and triploid flowers at 72 h after pollination (*χ* ^2^ = 7.5, *p* = 0.60). Analysis of covariance showed that pollen tube length at 24 and 48 h post-pollination was significantly influenced by ploidy and flower length of the female parent. Progeny of interploidy crosses was diploid and aneuploid; no triploid progeny were recovered from crosses using triploid parents. Mean genome sizes of offspring from each cross type ranged from 4.56 pg for 2x × 2x offspring to 5.17 pg for 3x × 3x offspring. Estimated ploidy of offspring ranged from 2x for 2x × 2x crosses to 2.4x for 3x × 3x crosses. Pollen stainability rates of flowering offspring using a modified Alexander's stain ranged from 69.6% to 76.4%.

## Introduction

Polyploidy—also known as whole genome duplication—represents a major mechanism of adaptation and speciation in plants (Grant, 1981; Ramsey and Schemske, 1998). It is widespread among plant families and an estimated 47 to 70% of angiosperm species are polyploid (Grant, 1981). Polyploidy has also been associated with changes to ornamental traits in plants. Increasing chromosome copy number leads to an increase in the number of each gene, a phenomenon known as “genetic redundancy”. The genetic redundancy present in polyploids enables them to undergo extensive genetic rearrangements that lead to the stable expression of novel traits (Kumari and George, 2008). Polyploids can serve as a bridge that allows interspecific hybridization between two species previously separated by ploidy differences and can be used to restore fertility to sterile hybrids (Lim et al., 2000). Polyploidization has also been used to develop sterile cultivars, enhance stress tolerance to drought, cold, herbicides, and poor soils, and enhance flower size, color, and other ornamental attributes (Takamura and Miyajima, 1996; Griesbach, 2000; Van Tuyl and Lim, 2003; Kumari and George, 2008).

*Hydrangea macrophylla* is one of the most economically important nursery crops worldwide, with U.S. sales of *Hydrangea* species topping $120,000,000 in 2014 (USDA-NASS, 2014). Hydrangeas, prized for their large showy flowers and lush green foliage, are sold for landscape use, patio and container use, and as cut flowers in the floral industry. Chromosome number within the genus ranges from 2n = 2x = 30 in *H*. *involucrata* Sieb. to 2n = 4x = 72 or 2n = 6x = 108 in *H. paniculata* Sieb. (Funamoto and Tanaka, 1988). All *H. macrophylla* cultivars tested to date are diploid (2*n*=2*x*=36) or triploid (2*n*=3*x*=54; Demilly et al., 2000; Cerbah et al., 2001; Zonneveld, 2004; Jones et al., 2007). Alexander (2017) showed that triploid hydrangeas had thicker stems, larger flowers, and larger stoma compared to full-sibling diploids. These triploids were meiotic polyploids produced using unreduced gamete breeding, where a source of n and 2n male gametes combined with meiotically normal female gametes to form both diploid and triploid full-sibling offspring (Bretagnolle and Thompson, 1995; Alexander, 2017). As observed in other species, triploids may also be obtained through somatic fusion or sexual hybridization between a diploid and tetraploid parent. Sexual hybridization between triploids and diploids has been used in a few, diverse ornamental species with fertile triploids like tulip (*Tulipa*) and pear (*Pyrus*) to generate genetic variability or new ploidy levels for interspecific hybridization (Cao et al., 2002; Marasek-Ciolakowska et al., 2014). It is unknown whether interploidy crosses between diploid and triploid hydrangeas can be used to develop triploid varieties, although a recent reconstruction of an *H*. *macrophylla* pedigree identified at least four putative interploidy crosses that produced high-quality diploid and/or triploid progenies (Hempel et al., 2018).

Fertility of triploids varies widely among plant species and is dependent on frequency and type of trivalents formed at metaphase I and the tolerance of gametes to aneuploidy (Singh, 1993). Stainable pollen for the diploid and triploid *H. macrophylla* ssp. macrophylla cultivars averaged 70% and 63%, respectively, and results of controlled pollinations indicate that triploid cultivars can produce viable seed (Jones et al., 2007). Breeding improvement using mixed ploidy levels requires the adoption of cytological methods to assess fertility of potential parents and suitability of potential cross combinations. Pollen staining is widely used to assess male fertility and pollen viability; however, pollen staining only assesses fertilization potential because not all stainable grains may actually be viable. Alexander (2019) showed that aniline-blue staining overestimated pollen viability by an average of 2.3x for *H*. *macrophylla* when compared to *in vitro* germination. Results of pollen germination using growth medium are considered more accurate, but still fail to account for pollen-pistil interactions. Using fluorescent microscopy to monitor pollen germination and growth of the pollen tube most closely simulates *in vivo* conditions (Kho and Baër, 1968; Atlagić et al., 2012). Further, visualization of pollen germination and pollen tube growth provides a measure of “cross” incompatibility among species or cultivars. The fruit of a hydrangea is a capsule containing dozens to hundreds of ovules that are capable of maturation into viable seeds (Hufford, 2001). Hybrid seed production is often low in *H*. *macrophylla*, possibly due to inbreeding in the cultivated gene pool (Wu and Alexander, 2019). Jones et al. (2007) reported from 0 to 20 seeds/capsule while other researchers have reported as little as 0–5 seeds/capsule resulting from crosses among *H. macrophylla* cultivars (Venturieri et al., 2017). Visualization of pollen germination and pollen tube growth allows for the differentiation between pre and post zygotic reproductive barriers which in turn influences the choice of plant breeding techniques (somatic hybridization or embryo culture) that may be used to mitigate incompatibility (Atlagić et al., 2012).

Interploidy hybridization which results in fertile progenies may generate new, desirable traits in established hydrangea cultivars or allow for the transfer of traits between hydrangea species. The objective of this study was to 1) compare pollen tube development, fruit formation, and seed viability among intra- and interploidy pollinations of *H. macrophylla*, and 2) evaluate the genome size and pollen viability of resultant progeny. Results will be used to identify reproductive barriers between ploidy levels and determine the efficacy of using triploids in *H. macrophylla* hybrid breeding. Progeny from interploidy hybridizations would be beneficial to expand the relatively narrow gene pool of cultivated hydrangea.

## Materials and Methods

### Plant Material

Three triploid *H. macrophylla* cultivars (Blaumeise, Kardinal, and Taube) and three diploid cultivars (Decatur Blue, Oakhill, and Zaunkoenig) were used as parents in this experiment. Ten 1 year-old clones per cultivar were used in this experiment. Clones were produced by rooting softwood cuttings in pine bark using intermittent mist and 1,500 ppm indole 3-butyric acid. Plants were grown indoors in 3-gallon containers under 56% shade and micro-irrigated using spray stakes. Growing media consisted of pine bark amended with 6.6 kg·m^–3^ 19N-2.1P-7.4K Osmocote Pro fertilizer (Scotts-Sierra Horticultural Products Co., Maryville, Ohio), 0.6 kg·m^–3^ Micromax (Scotts-Sierra Horticultural Products Co.), 0.6 kg·m^–3^ iron sulfate, and 0.2 kg·m^–3^ Epsom salts. Greenhouse temperatures were maintained at 24°C during the d and 20°C at night.

### Pollinations

Controlled pollinations were made following the method of Reed (2004). Briefly, pollen receptor flowers were emasculated 1–2 days before pollination and covered with breathable bags (DelStar, Inc., Middleton, DE). Pollen from donor flowers was collected in glass vials the day of pollination, pollen was applied to exposed receptor stigmas using a camel hair brush, and the bag was replaced. A new brush was used for each pollination. Five inflorescences on each plant were used for pollination. To reduce pollen contamination, only one male parent was used on each inflorescence. Six flowers were pollinated on each inflorescence. Three pollinated flowers were harvested for pollen tube measurements—one after 24 h, one after 48 h, and one after 72 h. The other three pollinated flowers were left to develop into capsules. Bags were removed after 1–2 weeks. For estimations of seed set and seed viability, approximately 30 flowers were pollinated for each cross.

### Pollen Staining and Pollen Tube Visualization

To estimate parental pollen viability, fresh pollen from a single flower was placed on a microscope slide using a camel-hair brush (n = 3 flowers per plant). A 30 μL drop of modified Alexander's stain (Alexander, 1969; Peterson et al., 2000; Atlagić et al., 2012) was pipetted onto the slide and a coverslip was applied. Slides were observed after 30 min at 10× magnification using an Olympus BX-60 compound microscope with an Olympus Q Color 5 digital camera for image capture. Percent stained pollen was calculated as: (number of stained pollen grains/total number of pollen grains) x 100%.

To analyze pollen tube growth, approximately 30 flowers were pollinated for each cross as above. Flowers were collected 24, 48, or 72 h after pollination and prepared for visualization. Briefly, pistils were removed from flowers and fixed in Carnoy I (3:1; ethanol:glacial acetic acid) for at least 24 h at room temperature. The fixative was removed by pipetting and pistils were rinsed twice for 10 min in 70% ethanol with gentle agitation. Fresh 70% ethanol was added to cover pistils and pistils remained in ethanol until visualization (between 24 h and 6 d). On the day of visualization, ethanol was removed and pistils were rinsed in deionized water, hydrolyzed in 8 N NaOH for 1 – 3 h, and rinsed again with deionized water. Fully hydrolyzed pistils appeared translucent. Pistils were placed in a petri dish containing decolorized aniline blue (0.1% aniline blue dissolved in 0.1 N K_3_PO_4_) for 1 h, removed to a microscope slide with one 30 uL drop of stain (2:1; decolorized aniline blue:glycerin), and a cover-slip was applied to spread the tissues. Slides containing stained pistils were visualized using a fluorescent microscope (BX-60; Olympus America, Inc.) equipped with a 100 W high-pressure Hg lamp and a U-MNV near ultraviolet (400–410 nm) filter. Magnification depended on size of the flower. After slide preparation, between 6 and 10 intact flowers were available for each cross at each time point. Data recorded for three to six flowers per cross included length of longest three pollen tubes (mm) and whether pollen tubes reached the ovary (Y/N). Density of pollen tubes in the ovaries was scored as follows: 0 = none, 1 = Few (1–10 tubes), 2 = Some (11–20 tubes), 3 = Many (≥20 tubes; i.e., too many to count). Total length of each style was recorded. A total of 279 pollinated flowers were visualized, measured, and used for further analysis.

### Seed Collection and Germination

Capsules were harvested November 2017 and stored at 4°C for 35–50 days. One capsule from each clone was harvested to determine average number of seeds per capsule. The seed from all 10 capsules of a cross (one capsule from each clone) was pooled to determine germination percentages. Up to 100 seeds of each full-sib family were sown in trays (1,020 flats) containing #1 starter mix and placed into a germinator on 4 January 2018. Germination conditions were 16 h light/8 h dark and 22°C. Flats were placed on trays and water was added to each tray when media in control flats appeared dry. Germination began ~7 d after sowing and was recorded for 28 days. Up to 30 seedlings per family were transferred to 2" pots. Seedlings were moved to 4" pots in April 2018, to 1.5 gal pots in July 2018, and were maintained in 1.5 gal pots in a greenhouse throughout the remainder of the experiment under the same conditions as the parent cultivars.

### Seedling Pollen Viability and Genome Size Determination

Flowering, pollen viability, and genome size of seedlings was measured April–June 2019. For each flowering seedling, pollen was collected and stained in the same manner as the parental plants. Percent stained pollen was calculated as: (number of stained pollen grains/total number of pollen grains) x 100%.

Up to 30 offspring per cross and each parent cultivar were sampled for genome size determination. Approximately 0.5 cm^2^ of growing leaf tissue of sample and standard were chopped for 30 to 60 s in a plastic petri dish containing 0.4 mL extraction buffer (Partec CyStain ultraviolet precise P Nuclei Extraction Buffer; Partec GMBH Muenster, Germany). The resulting extract was passed through a 30 μM filter into a 3.5-mL plastic tube to which was added 1.6 mL Partec CyStain ultraviolet precise P Staining Buffer containing the fluorochrome 4ʹ, 6-diamidino-2-phenylindole (DAPI). The relative fluorescence of the total DNA was measured for each nucleus using a Partec PA-1 ploidy analyzer (Partec GMBH, Muenster, Germany). Results were displayed as histograms showing the number of nuclei grouped in peaks of relative fluorescence intensity. For each sample, at least 3000 nuclei were analyzed revealing a single peak with a coefficient of variation (CV) less than 4.9%. Genome sizes were calculated as nuclear DNA content for unreduced tissue (2C) as: 2C DNA content of tissue = (mean fluorescence value of sample ÷ mean fluorescence value of standard) × 2C DNA content of standard. *Pisum sativum* L. “Ctirad” with a 2C content of 9.09 pg was used as the internal standard (Doležel and Bartoš, 2005). Ploidy and genome sizes are the averages of two subsamples per plant.

### Statistics

Pearson's chi-square test of association was used to determine the relationship between cross type and number of flowers with pollen tubes reaching the ovaries. Initial investigation of the data showed a strong correlation between style length and pollen tube length such that analysis of covariance (ANCOVA) with style length as a covariate was chosen over analysis of variance (ANOVA) to more accurately represent the influence of ploidy level. ANCOVA was used to partition variance in pollen tube length into sources attributable to female ploidy level, male ploidy level, and environment (error) with female style length as a covariate. Means for each ploidy level or cross were compared using Tukey's studentized range test with an *α* = 0.05 significance level. Data for all analyses were checked for normality and non-constancy of variance using Shapiro-Wilk's and Levene's tests, respectively. All data analysis was performed using SAS^®^ software, Version 9.4 of the SAS system for Microsoft (Copyright ^©^ 2013, SAS Institute Inc., Cary, NC, USA).

## Results

### Pollen Viability of Parents

**Figure 1:**
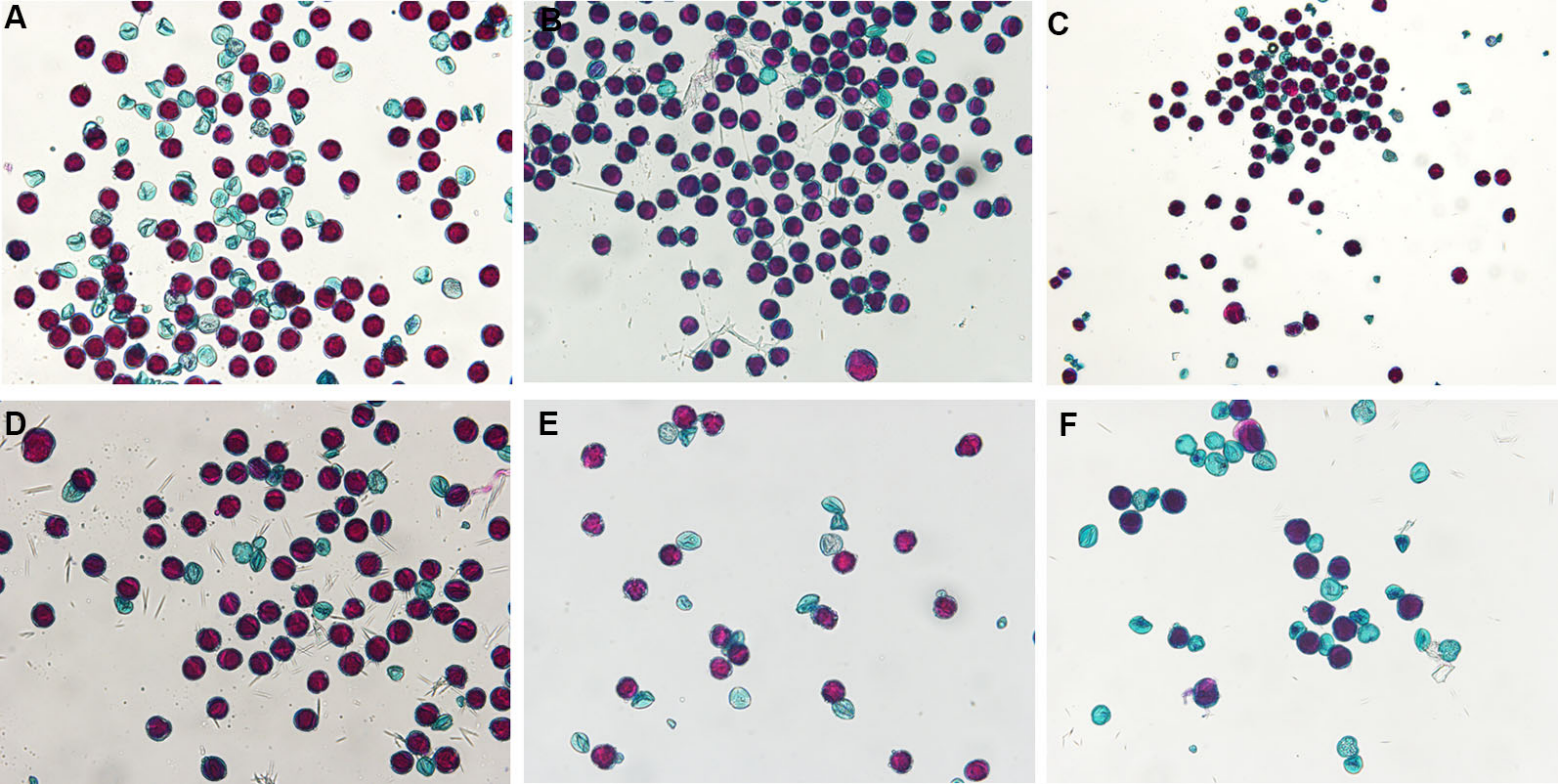
Stained pollen of *Hydrangea macrophylla* diploid cultivars Decatur Blue **(A)**, Oakhill **(B)**, and Zaunkoenig **(C)**, and triploid cultivars Blaumeise **(D)**, Nachtigall **(E)**, and Taube **(F)**. Fresh pollen was stained with modified Alexander's stain and observed after 30 min at 10× magnification using an Olympus BX-60 compound microscope with an Olympus Q Color 5 digital camera for image capture.

Percent stained pollen averaged 65 and 61% for diploid and triploid parent cultivars, respectively. One diploid parent, “Decatur Blue”, only had 44.1 ± 0.041% stainable pollen while the other diploid parents, “Oakhill” and “Zaunkoenig” produced 86.8 ± 0.064% and 65.2 ± 0.054% stainable pollen. Percent stained pollen for triploid parents was 75.3 ± 0.001%, 59.2 ± 0.026%, and 48.4 ± 0.12% for “Blaumeise”, “Nachtigall”, and “Taube”, respectively (**Figure 1**).

### Pollen Tube Growth

**Figure 2:**
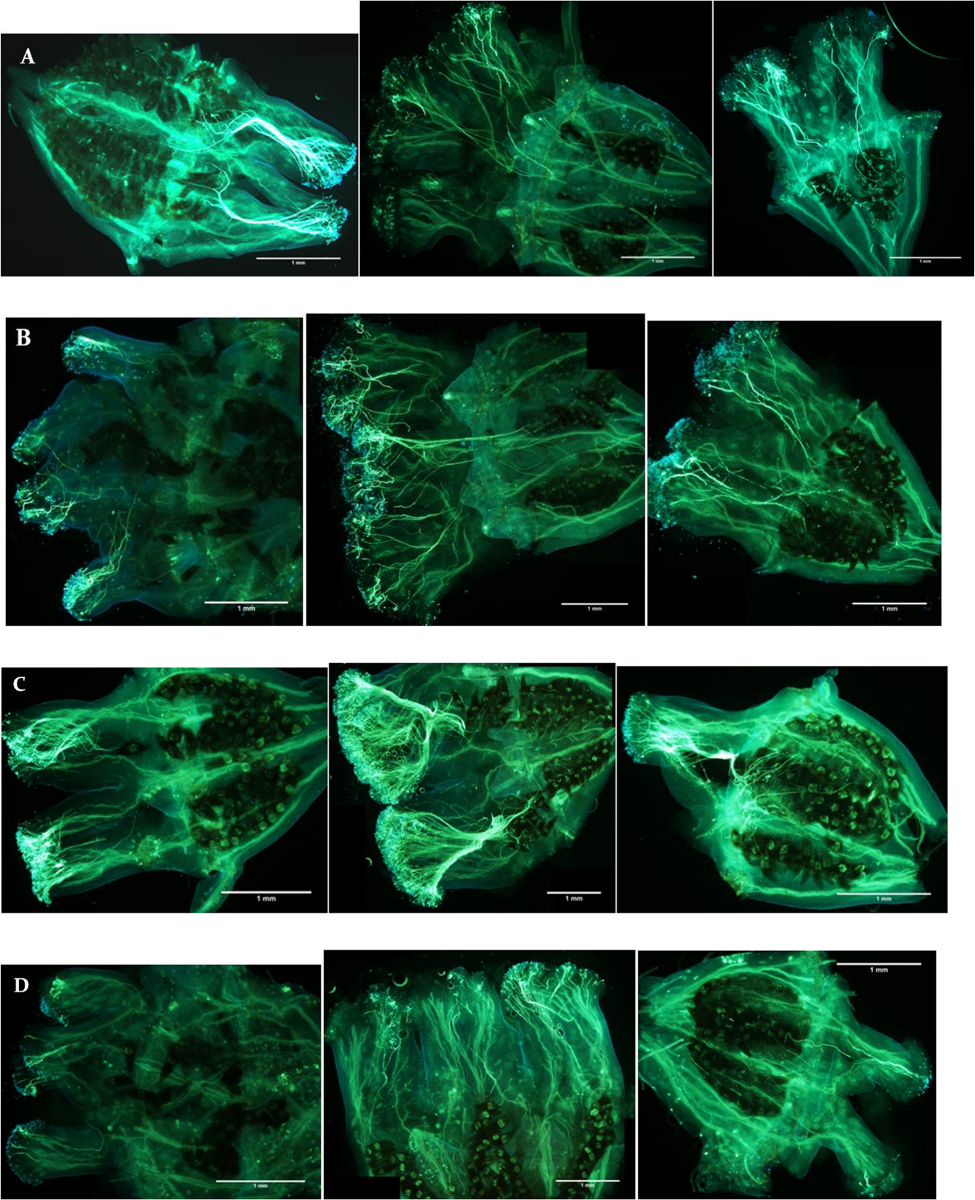
Stained pistils showing pollen tube growth from **(A)** 2x × 2x, **(B)** 2x × 3x, **(C)** 3x × 2x, and **(D)** 3x × 3x controlled crosses of *Hydrangea macrophylla* collected 24 h (left), 48 h (center), and 72 h (right) post-pollination. Crosses shown from top to bottom are: “Decatur Blue” x “Oakhill”, “Decatur Blue” x “Kardinal”, “Kardinal” x “Decatur Blue”, and “Taube” x “Kardinal”. Pistils were fixed, rinsed, hydrolyzed, stained with decolorized aniline blue, and placed on microscope slides. Slides were observed after 1 h using an Olympus BX-60 compound microscope with an Olympus Q Color 5 digital camera for image capture.

**Figure 3:**
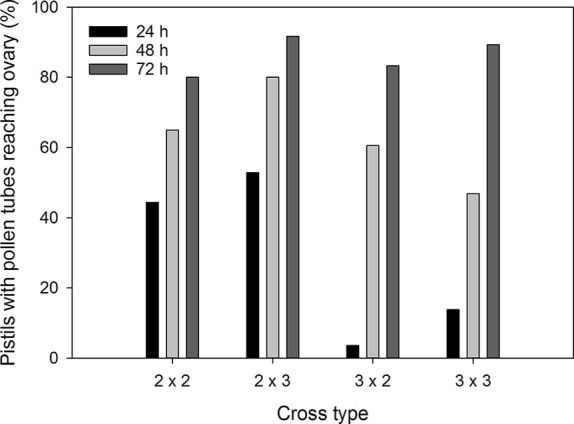
Mean percentage of *Hydrangea macrophylla* flowers with pollen tube reaching the ovaries at 24, 48, and 72 h post-pollination. 2 = 2*x* = diploid; 3 = 3*x* = triploid. Means are based on six crosses per cross type per time period. Error bars represent standard error of the mean. Pearson's chi-square values for association between cross type and percentage of flowers with pollen tubes reaching the ovaries were *χ* ^2^ = 30.6, *p* < 0.001 at 24 h, *χ* ^2^ = 26.5, *p* = 0.001 at 48 h, and *χ* ^2^ = 7.5, *p* = 0.60 at 72 h post-pollination.

Pollen germinated on the stigma of all flowers in this study, and all pairs of reciprocal crosses had some pollinations where pollen tubes reached the ovaries (**Figure 2**). There were significant associations between cross type and percentage of flowers with pollen tubes reaching the ovaries at 24 h (*χ* ^2^ = 30.6, *p* < 0.001) and 48 h (*χ* ^2^ = 26.5, *p* = 0.001) post-pollination. After 24 h, pollen tubes had reached the ovaries of diploid flowers in 44.4% of 2x × 2x crosses and 52.9% of 2x × 3x crosses. In contrast, pollen tubes reached the ovaries of triploid flowers in only 3.6% of 3x × 2x crosses and 13.8% of 3x × 3x crosses after 24 h (**Figure 3**). The trend continued at 48 h after pollination where pollen tubes reached the ovaries of diploid flowers in 65.0% of 2x × 2x crosses and 80.0% of 2x × 3x crosses. Pollen tubes reached the ovaries of triploid flowers in 60.6% of 3x × 2x crosses and 46.9% of 3x × 3x crosses after 48 h. There was no difference in percentage of flowers with pollen tubes reaching the ovaries in diploid and triploid flowers at 72 h after pollination (*χ* ^2^ = 7.5, *p* = 0.60).

Analysis of covariance showed that pollen tube length was significantly influenced by ploidy of the female parent and the style length of the female parent (**Table 1**). The 3x × 2x crosses had the longest pollen tubes at 24 and 48 h post-pollination (**Table 2**). The 3x × 3x pollinations had the shortest pollen tubes at 24 h and 2x × 2x and 3x × 3x crosses had the shortest pollen tubes at 48 h. There was no significant difference in pollen tube length among cross types by 72 h post-pollination. These comparisons were made adjusting for flower length, which accounted for most of the variation in pollen-tube length. Longer flowers tended to have longer pollen tubes; that is, pollen tubes grew until they reached the ovary regardless of the total size of the flower. Based on ANCOVA results, the influence of female ploidy disappeared after 48 h; flower length was the only significant source of variation in pollen tube length by 72 h post-pollination (**Table 1**).

**Table 1:** Analysis of covariance for sources of variation in pollen tube length for interploidy and intraploidy crosses of *Hydrangea macrophylla*.

Time	Source	DF	Mean Square	F Value	Pr > F
24 h	Female ploidy	1	1.9	3.7	0.0578
	Male ploidy	1	1.1	2.2	0.1433
	Flower length	1	12.6	24.0	<0.0001
	Error	95	0.5		
48 h	Female ploidy	1	4.6	4.1	0.0461
	Male ploidy	1	0.0	0.0	0.9597
	Flower length	1	34.0	30.0	<0.0001
	Error	96	1.1		
72 h	Female ploidy	1	1.9	2.3	0.1332
	Male ploidy	1	0.1	0.2	0.7012
	Flower length	1	69.5	84.9	<0.0001
	Error	79	0.8		

**Table 2:** Average pollen tube length (mm) and density (in italics) of pollen tubes from flowers of interploidy and intraploidy crosses of *Hydrangea macrophylla* collected 24, 48, and 72 h post-pollination.

Parents	Ploidy level of parents	Hours after pollination					
		24		48		72	
D × O^z^	2 × 2^y^	2.06 abc^x^	*0.6^w^*	2.97 bc	*2.2*	3.13 cd	*2.5*
O × D	2 × 2	1.31 bc	*0.0*	1.68 d	*0.5*	1.71 f	*1.0*
D × Z	2 × 2	2.52 a	*1.0*	3.01 bc	*1.5*	3.19 cd	*2.0*
Z × D	2 × 2	1.61 bc	*0.0*	1.72 de	*0.4*	1.89 ef	*1.5*
O × Z	2 × 2	1.15 cd	*1.4*	1.47 e	*1.9*	1.56 f	*2.5*
Z × O	2 × 2	2.24 ab	*0.2*	2.55 c	*0.5*	2.92 de	*1.0*
D × K	2 × 3	1.51 bc	*0.8*	2.37 cd	*1.0*	2.98 de	*1.7*
O × T	2 × 3	1.22 bc	*0.3*	1.88 d	*1.6*	2.16 def	*2.1*
Z × T	2 × 3	2.12 abc	*0.8*	2.90 bc	*1.0*	3.12 cd	*1.9*
D × T	2 × 3	2.32 ab	*1.0*	3.44 b	*2.0*	3.99 bc	*2.3*
O × K	2 × 3	1.76 abc	*0.5*	3.02 bc	*0.7*	3.17 cd	*1.1*
Z × B	2 × 3	1.04 cde	*0.8*	2.19 cd	*1.4*	2.68 def	*2.0*
K × Z	3 × 2	2.57 a	*0.0*	3.72 b	*2.0*	5.04 abc	*2.4*
K × O	3 × 2	1.69 abc	*0.1*	1.74 d	*0.4*	2.47 ef	*1.0*
K × D	3 × 2	2.27 ab	*0.0*	4.57 a	*2.1*	4.78 b	*2.5*
B × Z	3 × 2	2.42 a	*0.0*	3.51 b	*0.6*	4.26 bc	*1.4*
T × D	3 × 2	2.48 a	*0.0*	3.83 b	*1.7*	5.81 a	*2.3*
T × Z	3 × 2	1.95 bc	*0.0*	3.21 bc	*0.4*	5.12 ab	*1.0*
T × B	3 × 3	1.05 d	*0.0*	2.40 cd	*0.8*	3.91 bc	*2.2*
B × T	3 × 3	0.74 e	*0.0*	1.60 de	*1.1*	3.02 cd	*1.9*
B × K	3 × 3	1.20 cd	*0.0*	2.30 cd	*0.4*	4.13 bc	*1.5*
K × B	3 × 3	0.98 de	*0.0*	1.93 d	*0.5*	2.33 ef	*1.0*
K × T	3 × 3	2.25 ab	*0.0*	3.45 b	*0.6*	4.23 b	*2.0*
T × K	3 × 3	1.90 abc	*0.9*	2.27 cd	*1.8*	3.75 bc	*2.5*

z D, Decatur Blue; O, Oakhill; Z, Zaunkoenig; B, Blaumeise; K, Kardinal; T, Taube.

y 2, 2x, diploid; 3, 3x = triploid.

x Pollen tube lengths were analyzed *via* ANCOVA with female flower lengths as a covariate. Cross means were separated using Tukey's studentized range test. Means followed by the same letter were not significantly different at the *α* = 0.05 significance level. The longest three pollen tubes of three flowers were measured for each cross.

w Density of pollen tubes in the ovaries was scored as follows: 0 = none, 1 = Few, 2 = Some, 3 = Many.

### Seed Set, Germination, and Seedling Growth

Number of flowers pollinated for each cross ranged from 23 to 37. All cross types except 3x × 2x had at least one cross fail as defined by the absence of rounded capsules (**Table 3**). Many crosses had fully-formed capsules that contained no seed. For example, only 1 seed was obtained from the cross 3x × 3x cross “Kardinal” × “Taube” even though 11 capsules appeared mature. Average number of seeds per capsule ranged from 0.1 for the 3x × 3x cross “Kardinal” × “Taube” to 63.8 for its reciprocal, “Taube” × “Kardinal” (**Table 3**). Germination rate ranged from 74% for the 2x × 2x cross “Zaunkoenig” × “Decatur Blue” to 0% for two 2x × 3x, three 3x × 2x, and one 3x × 3x cross (**Table 3**).

**Table 3:** Percent fruit set, average number of seeds/fruit, seeds used for germination, germination rate, and six-month seedling survival of progenies resulting from interploidy and intraploidy crosses of *Hydrangea macrophylla*.

Parents	Ploidy level of parents	Fruit set (%)	Average number of seeds/fruit	Seeds used for germination	Germination (%)	Number (%) alive after six months
D × O^z^	2 × 2^y^	0.0	0.0	0	0.0	0 (0.0)
O × D	2 × 2	44.0	6.7	74	46.1	34 (100.0)
D × Z	2 × 2	46.7	4.6	65	50.8	32 (97.0)
Z × D	2 × 2	51.6	6.9	100	74.0	72 (97.3)
O × Z	2 × 2	41.2	52.4	100	61.0	61 (100.0)
Z × O	2 × 2	50.0	9.1	91	35.2	31 (96.9)
D × K	2 × 3	0.0	0.0	0	0.0	0 (0.0)
O × T	2 × 3	16.2	9.7	58	37.9	12 (54.5)
Z × T	2 × 3	18.4	10.3	62	8.0	0 (0.0)
D × T	2 × 3	0.0	0.0	0	0.0	0 (0.0)
O × K	2 × 3	35.0	0.9	6	0.0	0 (0.0)
Z × B	2 × 3	35.0	0.7	5	0.0	0 (0.0)
K × Z	3 × 2	78.0	1.5	17	0.0	0 (0.0)
K × O	3 × 2	100.0	15.4	100	21.0	18 (85.7)
K × D	3 × 2	71.4	5.0	100	15.0	14 (93.3)
B × Z	3 × 2	91.7	0.3	3	0.0	0 (0.0)
T × D	3 × 2	59.0	3.1	62	17.7	0 (0.0)
T × Z	3 × 2	38.5	0.8	4	0.0	0 (0.0)
T × B	3 × 3	100.0	9.0	100	6.0	0 (0.0)
B × T	3 × 3	0.0	0.0	0	0.0	0 (0.0)
B × K	3 × 3	0.0	0.0	0	0.0	0 (0.0)
K × B	3 × 3	85.7	8.9	100	8.0	1 (12.5)
K × T	3 × 3	64.7	0.1	1	0.0	0 (0.0)
T × K	3 × 3	23.5	63.8	100	10.0	1 (10.0)

^z^D, Decatur Blue; O, Oakhill; Z, Zaunkoenig; B, Blaumeise; K, Kardinal; T, Taube.

^y^2, 2x, diploid; 3 = 3x, triploid.

**Figure 4:**
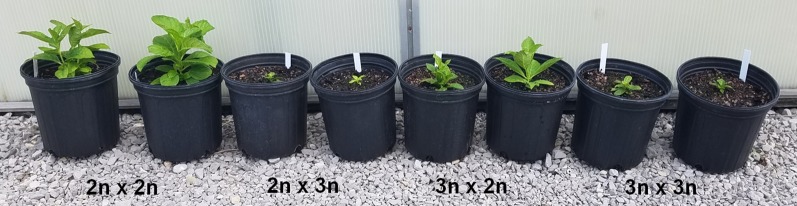
*Hydrangea macrophylla* seedlings from interploidy and interploidy crosses after five months of growth in a greenhouse under 56% shade at 24°C/20°C day/night and natural light. Photo taken 11 July 2018 in McMinnville, TN, USA.

Seedlings grew slowly after germination, especially those resulting from 2x × 3x and 3x × 3x crosses (**Figure 4**). Percent mortality for each cross type was calculated by adding the survival rates for each cross, dividing by the number of crosses, and subtracting from 100. Only crosses that produced seed were considered. By six months post-germination, mortality for 2x × 2x, 2x × 3x, 3x × 2x, and 3x × 3x seedlings was 1.8%, 45.5%, 10.5%, and 88.8%, respectively (**Table 3**).

### Genome Size and Pollen Viability of Seedlings

All six parental cultivars and from 2 to 30 seedlings from each cross type were analyzed for genome size (**Table 4**). Genome sizes of diploid parents measured approximately 4.5 pg while triploid cultivars ranged between 6.2 and 6.4 pg. Overall mean 1C genome size was 2.17 pg. Offspring ploidy levels were calibrated with genome sizes based on these representative cytotypes using the equation: mean estimated ploidy level (*x*) = mean 2C genome size ÷ mean 1C genome size (Beck and Ranney, 2014). Mean genome sizes of offspring from each cross type ranged from 4.56 for 2x × 2x offspring and 5.17 for 3x × 3x offspring. Estimated ploidy of offspring ranged from 2x for 2x × 2x crosses to 2.4x for 3x × 3x crosses (**Table 4**). DAPI stain, which we used, has a higher affinity for AT bases than propidium iodide (PI) stain which shows no base preference. Thus, the genome size estimates presented herein likely slightly overestimate the total nuclear DNA content of the *H. macrophylla* we tested (Zonneveld, 2004; Jones et al., 2007).

**Table 4:** Genome size and estimated ploidy for parent cultivars and offspring of *Hydrangea macrophylla* interploidy and intraploidy crosses.

Parent cultivar or cross type	N^z^	2C genome size (pg)	SD^y^	Min.^x^	Max.^w^	Estimated ploidy level (*x*)^t^
Parent cultivar						
Decatur Blue	1	4.54	0.05	–	–	2
Oakhill	1	4.59	0.01	–	–	2
Zaunkoenig	1	4.56	0.05	–	–	2
Blaumeise	1	6.24	0.03	–	–	3
Kardinal	1	6.39	0.01	–	–	3
Taube	1	6.22	0.12	–	–	3
Cross type^v^						
2 x 2	30	4.56	0.07	4.37	4.65	2
2 x 3	12	4.79	0.02	4.52	5.06	2.2
3 x 2	30	4.98	0.29	4.48	5.74	2.3
3 x 3	2	5.17	0.02	5.14	5.20	2.4

^z^Number of individuals sampled. Individual plant values are the mean of two subsamples.

^y^Standard deviation of genome size.

^x^Minimum individual plant genome size.

^w^Maximum individual plant genome size.

^v^2 = 2x = diploid; 3 = 3x = triploid.

^t^Mean estimated ploidy level (*extend*) = mean 2C genome size ÷ mean 1C genome size.

The number of offspring that produced flowers ranged from 0% for 3x × 3x crosses to 73% for 2x × 2x crosses (**Table 5**). Each of the three cross types with flowering offspring had some offspring that produced viable pollen. Sterility rates of flowering offspring ranged from 0% for 2x × 2x crosses to 50% for 2x × 3x crosses. Pollen stainability rates of flowering offspring ranged from 69.6% to 76.4% (**Table 5**). There were no significant differences in mean stainable pollen among cross types or between offspring and parent cultivars. However, comparisons of pollen viability between parents and offspring should be considered as rough estimates only as growth stage may influence pollen viability. The parent plants were one year-old cuttings taken from mature plants, and the offspring were grown from seed and measured the first time they flowered.

**Table 5:** Pollen stainability of offspring from intraploidy and interploidy crosses of *Hydrangea macrophylla*.

Cross type^y^	No. plants	Fertility		Pollen stainability^z^ (%)	
		No. (%) plants flowered	No. (%) sterile plants	Mean ± SD^x^	Range
2n × 2n	30	22 (73.3%)	0 (00.0%)	76.4 ± 0.09	(62.1–96.4)
2n × 3n	12	4 (33.3%)	2 (50.0%)	69.6 ± 0.06	(65.1–73.4)
3n × 2n	30	16 (53.3%)	2 (12.5%)	73.8 ± 0.13	(50.9–95.2)
3n × 3n	2	0 (00.0%)	–	–	–

^z^Fresh pollen was stained with modified Alexander's stain and observed after 30 min at 10× magnification using an Olympus BX-60 compound microscope.

^y^2, 2x = diploid; 3, 3x = triploid.

^x^Standard deviation.

## Discussion

### Pollen Tube Growth

Sympatric occurrences of two or more ploidy levels within a single morphologically defined species have been documented in many groups of vascular plants (Herben et al., 2016). Sympatry of different ploidy levels primarily arises through repeated formation of unreduced gametes by diploids (primary origin) or from secondary contact between previously allopatric diploid and polyploid populations (secondary origin; Petit et al., 1999). *H. macrophylla* shows both high rates of 2n gamete formation (Tränkner et al., 2019) and weak internal pre-zygotic reproductive barriers between ploidy levels as both diploids and triploids are nearly identical with respect to morphology, phenology, and likely habitat preferences (McClintock, 1957). The current study provides insight into a potential pre-zygotic barrier relating to pollen tube growth, where tubes of pollen from triploid parents grew more slowly than tubes of pollen from diploid parents, regardless of the ploidy level of the female parent. This may be because tubes from aneuploid pollen grains grow more slowly than tubes from haploid (n) gametes or diploid (2n) gametes, and all of the pollen produced by triploids in this study appeared to be aneuploid based on progeny genome sizes.

### Seed Set, Germination, and Seedling Growth

Triploid block is a well-studied post-zygotic reproductive barrier that operates in the endosperm, a terminal tissue surrounding the embryo (Brink and Cooper, 1947; Ramsey and Schemske, 1998; Köhler et al., 2010). In crosses between diploid individuals, a haploid pollen grain combines with a haploid egg cell to form a diploid embryo, while the surrounding endosperm tissue is triploid. In interploidy hybridizations, the parental genome contributions are altered, which affects endosperm development and the formation of viable seeds (Schatlowski and Köhler, 2012). Jones et al. (2007) reported that mean number of seed per cross and percent seed germination were higher when triploid cultivars were used as the pistillate (female) rather than the staminate (male) parent in controlled crosses. Results herein indicated that not only does parental ploidy influence seed set and seed germination, but that parental influence continues throughout plant growth and flowering. Interploidy crosses with triploids as opposed to diploids as the female parent showed higher seed set, higher germination rates, higher survival rates, and higher rates of flowering. After double-fertilization, the endosperm of a 3x × 2x cross will have a multiple of a complete set of chromosomes (4x) while the endosperm of a 2x × 3x cross will contain an imbalanced chromosome complement (3.5x). These data support the idea that endosperm imbalance is a major post-zygotic barrier in *H. macrophylla*. Another major post-zygotic reproductive barrier is fitness of offspring (Herben et al., 2016). The current study showed slow growth and low survival of offspring resulting from interploidy crosses, supporting the primary origin hypothesis of triploid *H. macrophylla*. However, as proportion of unreduced gametes (determined by pollen visualization and hybridization experiments) is genotype-specific (Herben et al., 2016; Alexander, 2017) more triploid cultivars should be screened for unreduced gamete production.

Sexual hybridization among ploidy levels has played a key role in breeding improvement for a large number of woody ornamental species where crosses between tetraploid and diploid parents are used to produce sterile triploid hybrids with unique traits (Kumari and George, 2008). Breeders often face strong or absolute sterility of triploid hybrids which prevents further breeding using triploid individuals. In the case of *H*. *macrophylla*, triploids have nearly identical fertility to diploids and that fertility is cultivar specific (Jones et al., 2007; Alexander, 2017). All three triploid cultivars used as parents in this study had a higher fertility (as measured by percent stainable pollen) than the least fertile diploid parent. Fertility of triploids determined by staining is corroborated by the presence of offspring in this and other studies, and by the results of pollen tube growth presented herein. The offspring were also fertile such that progeny with unique traits could be used for future breeding. However, the high number of aneuploid progeny indicates that aneuploid gametes appear viable using the staining method described herein.

### Possible Origin of Triploid Cultivars

Progeny of interploidy crosses was diploid and aneuploid; no triploid progeny was recovered from crosses using triploid parents. Based on the genome size of surviving progeny, it can be inferred that few 1x and many aneuploid gametes were produced by triploids, with no evidence of successful pollination by 2n gametes that would be necessary to produce triploid offspring. Success of using triploid parents to produce triploid progeny varies across species. The results of Cao et al. (2002) indicate that triploids were not useful in polyploid breeding of pear, while high numbers of triploids were obtained in 2x × 3x crosses of hybrid tulips (Marasek-Ciolakowska et al., 2014). Crosses between pentaploid and tetraploid *H. paniculata* tended to produce progeny with genome sizes within or near the tetraploid range, indicating a bias towards euploidy/isoploidy (Beck and Ranney, 2014). While no triploid *H*. *paniculata* were available for comparison, it can be inferred that the pentaploids produced aneuploid and 1x gametes, similar to the triploid *H*. *macrophylla* in the current study. In both cases, the polyploid hydrangea of uneven ploidy (i.e., 3x and 5x) failed to produce offspring reflective of the unreduced number of the closest lower ploidy (i.e., 2n gametes for triploids and 4n gametes for pentaploids).

Several hundred *H. macrophylla* cultivars were bred in the twentieth century based on seven or eight different plants that were brought to Europe from plant explorations to Japan and China in the 18^th^ and 19^th^ centuries (Bertrand, 2000). The origin of triploid *H. macrophylla* cultivars is unclear. Several *H. macrophylla* triploids were produced by a few breeders. Two German breeders, H. Schadendorff and F. Matthes, were responsible for six triploid cultivars, whereas another six were released by the Federal Research Institute for Horticulture in Switzerland (Mallet, 1994; Van Gelderen and Van Gelderen, 2004). It is not known whether these and other European breeders had access to tetraploid forms of *H. macrophylla* that were not included in previous ploidy analyses (Demilly et al., 2000; Cerbah et al., 2001; Zonneveld, 2004) or if they were using parental stocks that produced unreduced gametes. An additional possibility, considering the fertility of the triploids, is that a triploid parent may have been used in the breeding of additional triploids. Hempel et al. (2018) suggested that sexual hybridization between diploids and triploids was present in the pedigree of many well-known diploid and triploid cultivars. In the current study, controlled crosses between triploid and diploid *H*. *macrophylla* plants resulted in aneuploid offspring with differing chromosome numbers and poor growth compared to their parent cultivars. Therefore, the hypothesis of interploidy crosses as the source of triploid cultivars was not supported by this research, though it cannot be ruled out as a possibility. Additionally, high rates of unreduced gamete formation have been documented in *H. macrophylla*. An F1 mapping population from two diploid parents contained 103 diploids and 317 triploids (Tränkner et al., 2019). Alexander (2017) showed that controlled crosses using a cultivar known to produce unreduced male gametes resulted in 97% triploid offspring in the progeny. Considering the difficulty of obtaining interploidy crosses combined with their low survival rates, it appears that unreduced gamete breeding is the most suitable method for producing marketable triploids and is the most likely source of triploid *H. macrophylla* cultivars.

In conclusion, interploidy crosses among *H. macrophylla* diploid and triploid cultivars are associated with genetically unstable progeny. Viable seed from controlled interploidy crosses is difficult to obtain, survival is low, and progeny show poor growth and ornamental quality compared to their parent cultivars. Using triploid plants as the maternal parent improves success. *H. macrophylla* seems highly tolerant to aneuploidy and several aneuploid plants showed unique phenotypes and flowers with stainable pollen. The progeny from interploidy hybridizations have broad potential in breeding programs to provide genetic and phenotypic variability for the production of novel varieties.

## Data Availability Statement

The datasets generated for this study are available on request to the corresponding author.

## Author Contributions

LA conceived, designed, and supervised the experiment, analyzed the data, and wrote the manuscript.

## Conflict of Interest

The author declares that the research was conducted in the absence of any commercial or financial relationships that could be construed as a potential conflict of interest.

## References

[B1] Alexander M. P. (1969). Differential staining of aborted and non-aborted pollen. Biotech. Histochem. 44, 117–122. 10.3109/10520296909063335 4181665

[B2] Alexander L. (2017). Production of triploid *Hydrangea macrophylla via* unreduced gamete breeding. HortScience 52, 221–224. 10.21273/HORTSCI11358-16

[B3] Alexander L. (2019). Optimizing pollen germination and pollen viability estimates for *Hydrangea macrophylla*, *Dichroa febrifuga*, and their hybrids. Sci. Hortic. 246, 244–250. 10.1016/j.scienta.2018.11.008

[B4] Atlagić J. Terzic. S. Marjanović -Jeromela A. (2012). Staining and fluorescent microscopy methods for pollen viability determination in sunflower and other plant species. Ind. Crops Prod. 35, 88–91. 10.1016/j.indcrop.2011.06.012

[B5] Beck W. T. Ranney T. G. (2014). Ploidy levels and interploidy hybridization in panicle hydrangea (*Hydrangea paniculata*). Proc. So Nurs. Ass. Res. Conf. 59, 181–187.

[B6] Bertrand H. (2000). Management and knowledge of the *Hydrangea* collection of angers: morphological characters and data analysis. Acta Hortic. 508, 173–178. 10.17660/ActaHortic.2000.508.22

[B7] Bretagnolle F. Thompson J. D. (1995). Gametes with the somatic chromosome number: mechanisms of their formation and role in the evolution of autopolyploid plants. New Phytol. 129, 1–22. 10.1111/j.1469-8137.1995.tb03005.x 33874422

[B8] Brink R. A. Cooper D. C. (1947). The endosperm in seed development. Bot. Rev. 13, 423–477. 10.1007/BF02861548

[B9] Cao Y. Huang L. Shuling L. Yang Y. (2002). Genetics of ploidy and hybridized combination types for ploidy breeding in pear. Acta Hortic. 587, 207–210. 10.17660/ActaHortic.2002.587.24

[B10] Cerbah M. Mortreau E. Brown S. Siljak-Yakovlev S. Bertrand H. Lambert C. (2001). Genome size variation and species relationships in the genus H*ydrangea* . Theor. Appl. Genet. 103, 45–51. 10.1007/s001220000529

[B11] Demilly D. Lambert C. Bertrand H. Cadic A. (2000). Diversity of nuclear DNA contents of *Hydrangea* . Acta Hortic. 508, 281–284. 10.17660/ActaHortic.2000.508.45

[B12] Doležel J. Bartoš J. (2005). Plant DNA flow cytometry and estimation of nuclear genome size. Ann. Bot. (Lond) 95, 99–110. 10.1093/aob/mci005 PMC4246710 15596459

[B13] Funamoto T. Tanaka R. (1988). Karyomorphological studies in some taxa of *Hydrangea* from Japan. La Kromosomo 49, 1583–1594.

[B14] Grant V. (1981). Plant Speciation. 2nd edition (New York: Columbia Univ. Press).

[B15] Griesbach R. J. (2000). Day lilies of the field. (Chicago, Illinos: University of Chicago Magazine), 92, 6.

[B16] Hempel P. Hohe A. Tränkner C. (2018). Molecular reconstruction of an old pedigree of diploid and triploid *Hydrangea macrophylla* genotypes. Front. Plant Sci. 9, 429. 10.3389/fpls.2018.00429 29720985 PMC5915539

[B17] Herben T. Trávnáček P. Chrtek J. (2016). Reduced and unreduced gametes combine almost freely in a multiploidy system. Perspect. Plant Ecol. Evol. Syst. 18, 15–22. 10.1016/j.ppees.2015.12.001

[B18] Hufford L. (2001). Ontogeny and morphology of the fertile flowers of *Hydrangea* and allied genera of tribe Hydrangeeae (Hydrangeaceae). Bot. J. Linn. Soc 137, 139–187. 10.1006/bojl.2001.0475

[B19] Jones K. D. Reed S. M. Rinehart T. A. (2007). Analysis of ploidy level and its effects on guard cell length, pollen diameter, and fertility in *Hydrangea macrophylla* . HortScience 42, 483–488. 10.21273/HORTSCI.42.3.483

[B20] Köhler C. Mittelsten Scheid O. Erilova A. (2010). The impact of the triploid block on the origin and evolution of polyploid plants. Trends Genet. 26, 142–148. 10.1016/j.tig.2009.12.006 20089326

[B21] Kho Y. O. Baër J. (1968). Observing pollen tubes by means of fluorescence. Euphytica 17, 298–302. 10.1007/BF00021224

[B22] Kumari I. P. George T. S. (2008). Application of polyploidy breeding in ornamentals. Curr. Biotica 2, 121–145.

[B23] Lim K. B. Chung J. D. Van Kronenburg B. C. E. Ramanna M. S. De Jong J. H. Van Tuyl J. M. (2000). Introgression of *Lilium rubellum* Baker chromosomes into *L. longiflorum* Thunb: a genome painting study of the F1 hybrid, BC1 and BC2 progenies. Chromosome Res. 8, 119–125. 10.1023/A:1009290418889 10780700

[B24] Mallet C. (1994). Hydrangeas: Species and cultivars, Vol. 2 (Varengeville: Centre d'Art Floral).

[B25] Marasek-Ciolakowska A. Xie S. Arens P. van Tuyl J. (2014). Ploidy manipulation and introgression breeding in Darwin hybrid tulips. Euphytica 198, 389–400. 10.1007/s10681-014-1115-3

[B26] McClintock E. (1957). A monograph of the genus *Hydrangea* . Proc. Calif. Acad. Sci. 29, 147–256.

[B27] Peterson R. Slovin J. P. Chen C. (2000). A simplified staining method of aborted and non-aborted pollen grains. Intl. J. Plant Biol. 1, e13. 10.4081/pb.2010.e13

[B28] Petit C. Bretagnolle F. Felber F. (1999). Evolutionary consequences of diploid–polyploid hybrid zones in wild species. Trends Ecol. Evol. 14, 306–311. 10.1016/S0169-5347(99)01608-0 10407427

[B29] Ramsey J. Schemske D. W. (1998). Pathways, mechanisms, and rates of polyploid formation in flowering plants. Annu. Rev. Ecol. Syst. 29, 467–501. 10.1146/annurev.ecolsys.29.1.467

[B30] Reed S. (2004). Self-incompatibility and time of stigma receptivity in two species of *Hydrangea* . HortScience 39, 312–315. 10.21273/HORTSCI.39.2.312

[B31] Schatlowski N. Köhler C. (2012). Tearing down barriers: understanding the molecular mechanisms of interploidy hybridizations. J. Exp. Bot. 63, 6059–6067. 10.1093/jxb/ers288 23105129

[B32] Singh R. J. (1993). Plant Cytogenetics. (Boca Raton: CRC Press).

[B33] Takamura T. Miyajima I. (1996). Colchicine induced tetraploids in yellow flowered cyclamens and their characteristics. Sci. Hortic. 65, 305–312. 10.1016/0304-4238(96)00896-5

[B34] Tränkner C. Kruger J. Wanke S. Naumann J. Wenke T. Engel F. (2019). Rapid identification of inflorescence type markers by genotyping-by-sequencing of diploid and triploid F1 plants of *Hydrangea macrophylla* . BMC Genet. 20, 60. 10.1186/s12863-019-0764-6 31337331 PMC6651981

[B35] USDA-NASS (2014). 2014 Census of Horticulture Specialties. National agricultural statistics service, U.S. department of agriculture. Accessed from: http://www.agcensus.usda.gov/Publications/2012.

[B36] Van Gelderen C. J. Van Gelderen D. M. (2004). Encyclopedia of Hydrangeas. (Portland: Timber Press).

[B37] Van Tuyl J. M. Lim K. B. (2003). Interspecific hybridization and polyploidization as tools in ornamental plant breeding. Acta Hortic. 612, 13–22. 10.17660/ActaHortic.2003.612.1

[B38] Venturieri G. A. Nesi B. Lazzereschi S. Pecchiolo S. Burchi G. (2017). Development of pollination and *in vitro* germination techniques to improve the hybridization in *Hydrangea* spp. Adv. Hortic. Sci. 31, 45–51. 10.13128/ahs-20725

[B39] Wu X. Alexander L. (2019). Genetic diversity and population structure analysis of *Hydrangea macrophylla* using genotyping-by-sequencing (GBS). J. Amer. Soc Hortic. Sci. 144, 257–263. 10.21273/JASHS04683-19

[B40] Zonneveld B. J. M. (2004). “Genome size in *Hydrangea* ,” in Encyclopedia of Hydrangeas. Eds. van Gelderen C. J. van Gelderen D. M. (Portland, OR: Timber Press), 245–250.

